# Safety and immunogenicity of human papillomavirus vaccination in juvenile patients with rheumatic diseases

**DOI:** 10.1186/1546-0096-9-S1-O41

**Published:** 2011-09-14

**Authors:** MW Heijstek, N Groot, M Scherpenisse, C Tacke, G Berbers, F van der Klis, NM Wulffraat

**Affiliations:** 1 University Medical Center Utrecht, Wilhelmina Children’s Hospital, Utrecht, The Netherlands; 2 Centre for Infectious Disease Control Netherlands, National Institute of Public Health and the Environment, Bilthoven, the Netherlands

## Aim

To assess the safety and immunogenicity of the bivalent HPV vaccine (Cervarix) in patients with Juvenile Idiopathic Arthritis (JIA), Systemic Lupus Erythematosus (SLE) and Juvenile Dermatomyositis (JDM).

## Methods

A prospective controlled observational trial was initiated in female patients and healthy controls aged 12-18 years. Patients and controls were vaccinated (0, 1, and 6 months) and followed for 12 months. HPV16 and 18-specific antibody concentrations were measured using a fluorescent microspheres-based multiplex immuno-assay after 12 months. Adverse events (AE’s) 2 weeks after each vaccination were systematically documented in diaries. In patients, medication use was monitored and disease activity was measured by the Juvenile Arthritis Disease Activity Score of 27 joints (JADAS-27), SLE Disease Activity Index, or the Childhood Myositis Assessment Scale.

## Results

58 patients (45 JIA, 8 SLE, 5 JDM) and 51 healthy controls are currently included, patients are still being enrolled. The percentage of patients responding to vaccination (response rate) was similar in patients and controls. However, HPV16 antibodies were lower in JIA (5119 LU/ml, p=0.037), SLE (1461 LU/ml, p=0.002) and JDM patients (3233 LU/ml, p=0.262) compared with healthy controls (8799 LU/ml). HPV18 antibodies were similar in JIA patients and controls (2764 LU/ml versus 2522 LU/ml, p=0.732); SLE (730 LU/ml, p=0.030) and JDM patients (1147 LU/ml, p=0.384) showed markedly lower antibody levels. MTX-use did not lower vaccine responses.

The median JADAS-27 did change considerably between visits (1.9-2.5), but JADAS-27 was significantly elevated at 7 months (p=0.001) due to one outlier. SLE and JDM disease activity remained similar. AE’s were similar in patients and controls.

## Conclusion

Cervarix was safe in girls with rheumatic diseases. The response rates were similar in patients and healthy controls, whereas antibody concentrations were markedly lower in SLE and JDM patients.

